# Supplementary material for: Cryo-EM structure of aerolysin variants reveals a novel protein fold and the pore-formation process
Source: Nat Commun. 2016 Jul 13;7:12062. doi: 10.1038/ncomms12062 (PMC4947156; doi:10.1038/ncomms12062)
Supplement: Supplementary Information — Supplementary Figures 1-10, Supplementary Note 1 and Supplementary References [file ncomms12062-s1.pdf]

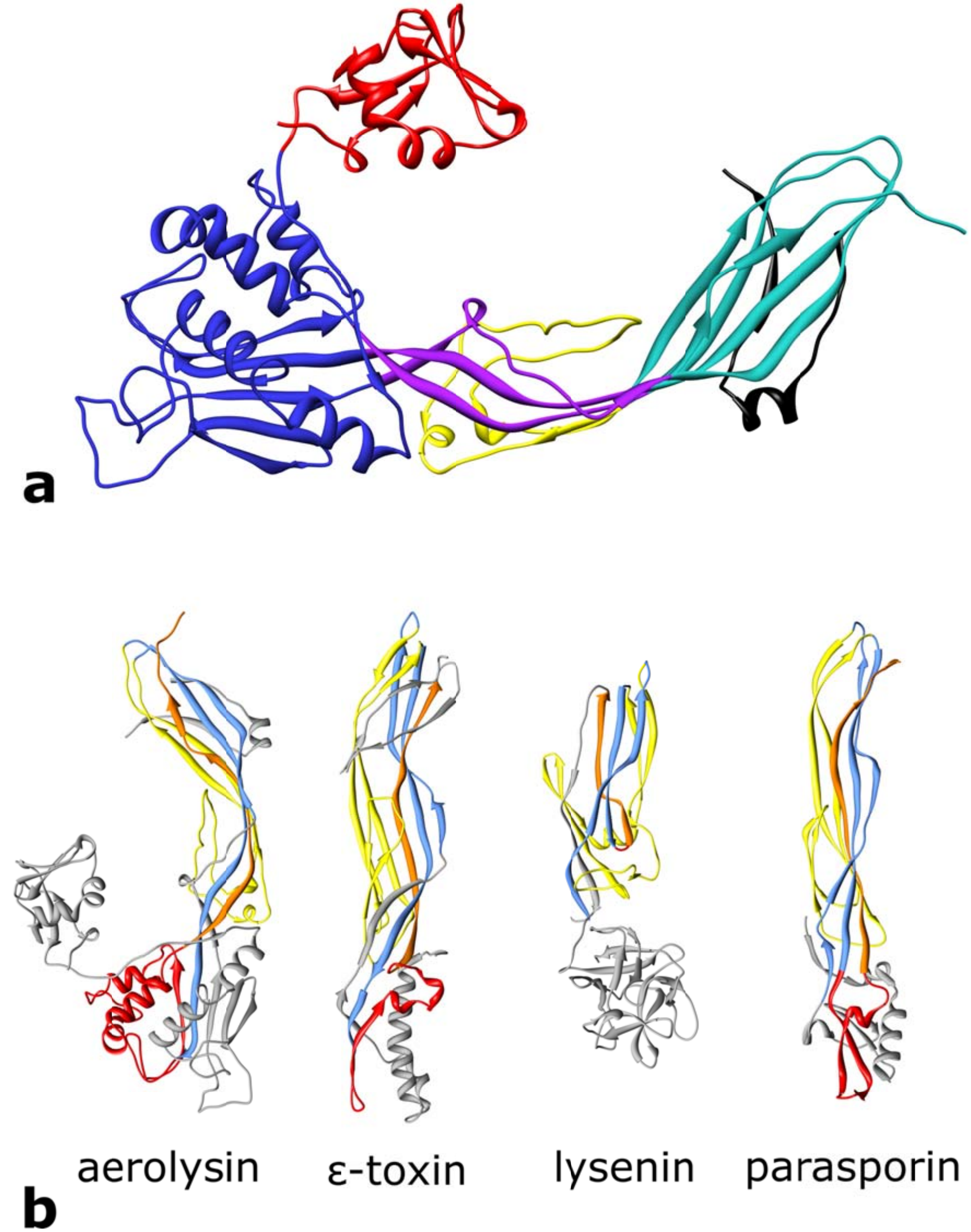

Supplementary Figure 1: soluble form structure of aerolysin family members

a. Structure of the soluble form of proaerolysin (PDB 1PRE) divided in structural domains. Domain 1 in red, domain 2 in blue, domain 3 in purple, domain 4 in turquoise. The prestem loop is shown in yellow whilst the C-terminal activation peptide (CTP) is shown in black. **b.** Aerolysin and aerolysin family members colour coded to highlight a common structural motif adopted from Szeczesny et. al[1]. The extended aerolysin family is characterised by a common structural motif which can be divided into a variable loop usually containing the binding side (red), two highly conserved β-strands (blue), an insertion loop (yellow) and weakly conserved β-strand (orange).

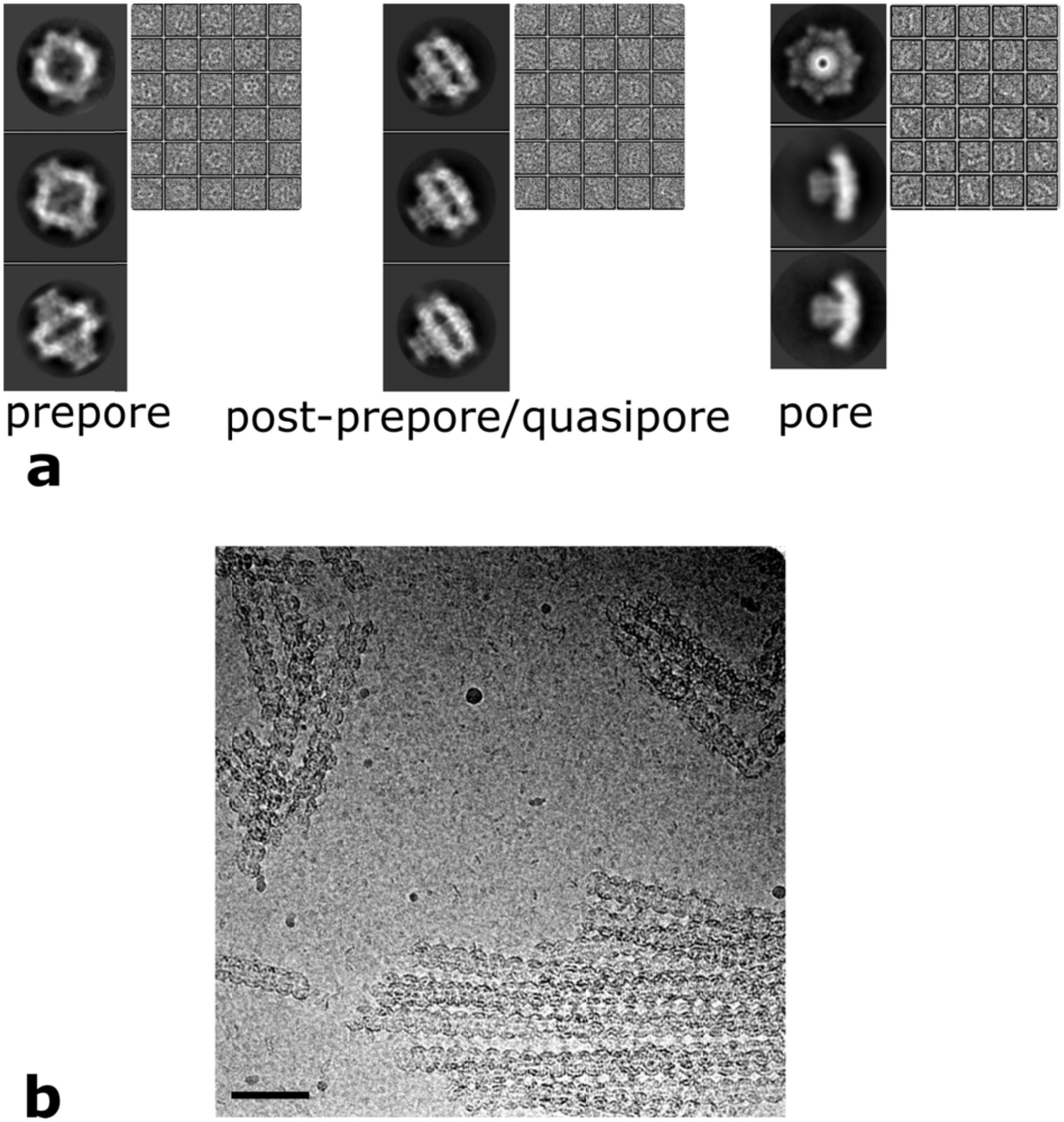

Supplementary Figure 2: Particles, class averages, and micrograph
a. Class averages and “shiny” particles from the prepore, postprepore/quasipore and pore (in LMNG micelles) datasets. Box size for prepore and postprepore/quasipore is 268 Å and 252 Å for the pore. **b.** Wild type aerolysin shows some aggregation even in the presence of detergent (scale bar: 100nm).

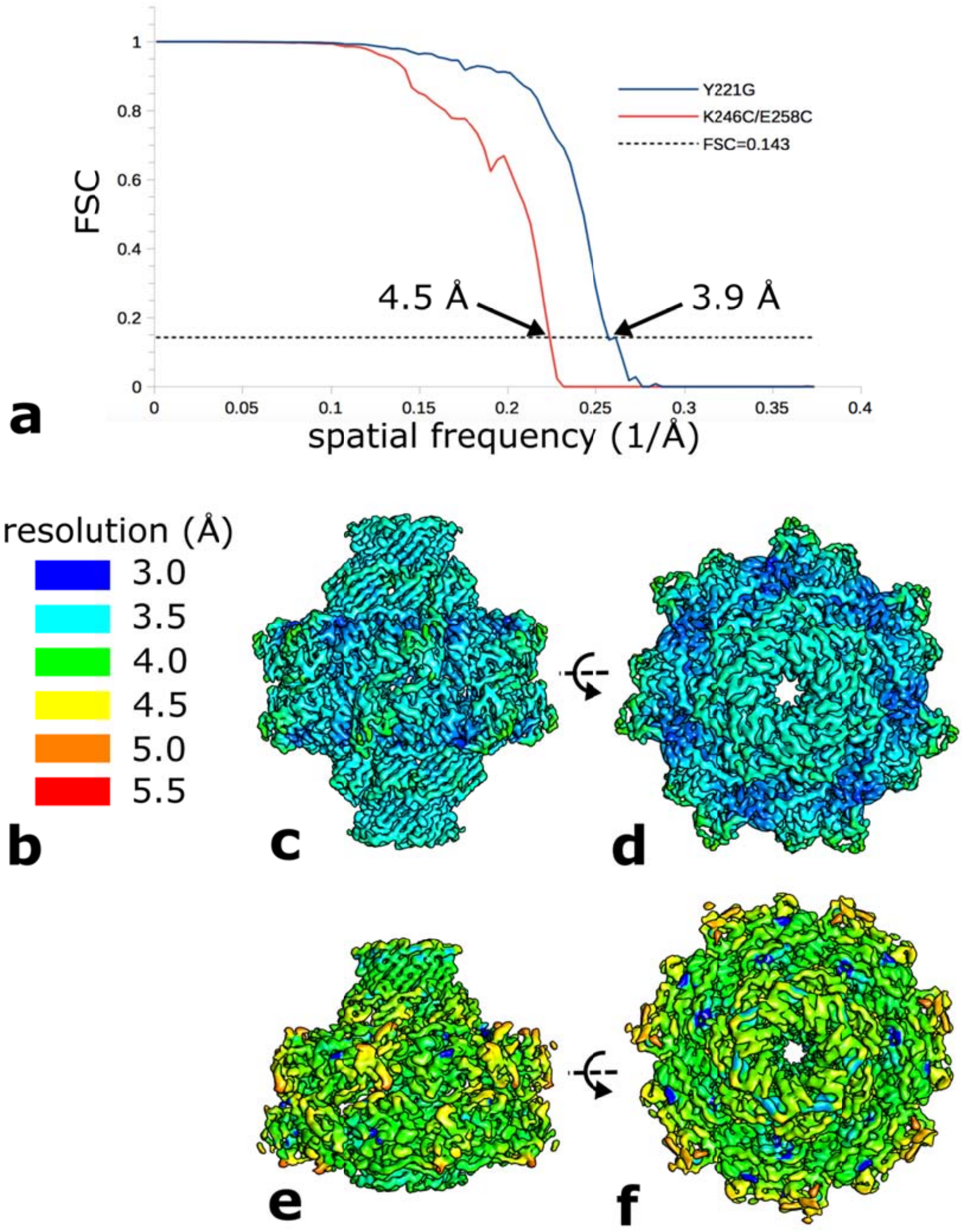

Supplementary Figure 3: Cryo-EM maps resolution estimation

Cryo-EM maps resolution estimation. **a**, Masking-effect-corrected gold standard FSC curves of the prepore (blue) and post- and quasipore (red) maps. The horizontal dashed line indicates the 0.143 cutoff used to estimate the global resolution of the two maps at 3.9 Å and 4.5 Å, respectively. **b**, gradient colour scale used for local resolution representation. **cd,** Side and top views of the prepore cryo-EM map colour-coded according to local resolution as in **b**. **ef**, Side and top views of the post-prepore and quasipore cryo-EM map colour coded as in **b**. **g,** Gold standard FSC curves for the wild-type aerolysin pore in detergent.

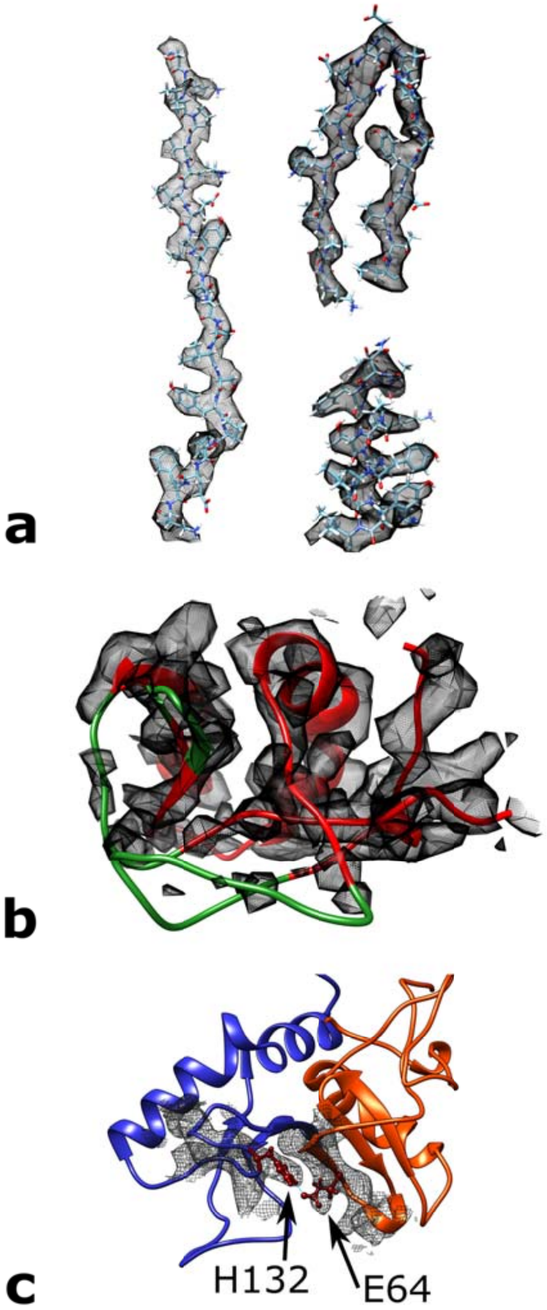

Supplementary Figure 4: Cryo-EM map resolution highlights
Cryo-EM map resolution highlights. **a**, Local level of detail in the prepore cryo-EM map showing residues 289 to 309 belonging to domains 3 and 4 (left), 198 to 219 belonging to domain 4 (top right) and 108 to 123 belonging to domain 2 (bottom right) as sticks with the corresponding density shown in mesh representation. Bulky side chain densities are clearly resolved in the cryo-EM map. **b**, Structure of the domain 1 (in red) docked in the corresponding density(mesh). The receptor binding loops (in green) are flexible and unresolved (L11-K22, L50-W54 and G68-N72). **c**, Inter-subunit hydrogen bond H132-E64. Part of one subunit ribbon diagram is drawn in blue and part of the next subunit is in red. A portion of the prepore feature-enhanced map around H132 and E64 is displayed in mesh.

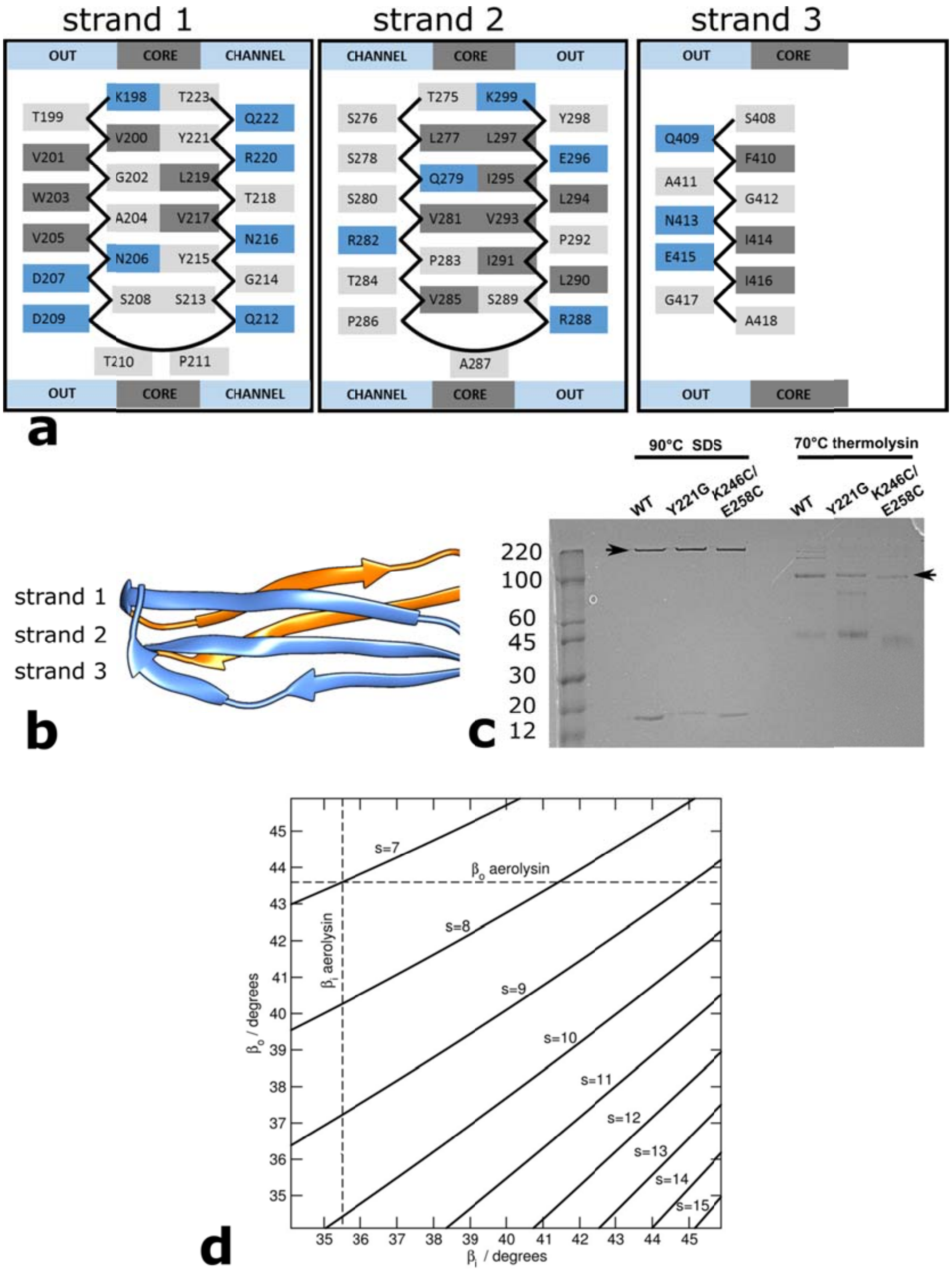

Supplementary Figure 5: Concentric β-barrel fold responsible for the stability and stoichiometry of the aerolysin oligomer

Concentric β-barrel fold responsible for the stability and stoichiometry of the aerolysin oligomer. **a**, Schematics of the residues involved in the formation of the concentric β-barrel fold. Hydrophobic residues are shown in dark grey, uncharged/indifferent residues in light grey whilst charged residues are in light blue. **b**, structure of one subunit of the β-barrel fold showing the position of the 3 strands referred to in **a**. **c**, SDS-PAGE of aerolysin wild-type (lanes 1 and 4), Y221G (lanes 2 and 5) and K246C/E258C (lanes 3 and 6) oligomers. Following proteolytic activation and oligomerisation, the sample was heated to 90° in presence of sample buffer (including 2% SDS) for 5 minutes and loaded on the gel (lanes 1-3). Most of the

oligomeric proteins are not denatured (left arrow). Oligomeric aerolysin was incubated at 70 °C in the presence of thermolysin for 10 minutes followed by addition of sample buffer and loading on the gel (lanes 6-8). These lanes show a similar band pattern to those published in[2], indicating that most of the proteins are only partially digested heptamers (right arrow). **d**, The outer β-barrel chain tilt angle is displayed as function of the inner β-barrel chain tilt angle for a range of aerolysin stoichiometry (*s*) according to equation 1 (see Methods). The measured values for aerolysin inner and outer β-barrel chain tilt angles are displayed with dashed lines. Only the acceptable range of chain tilt angles as defined in Methods is represented (35 to 45°). This analysis shows that the smallest possible aerolysin-like protein oligomer is a heptamer.

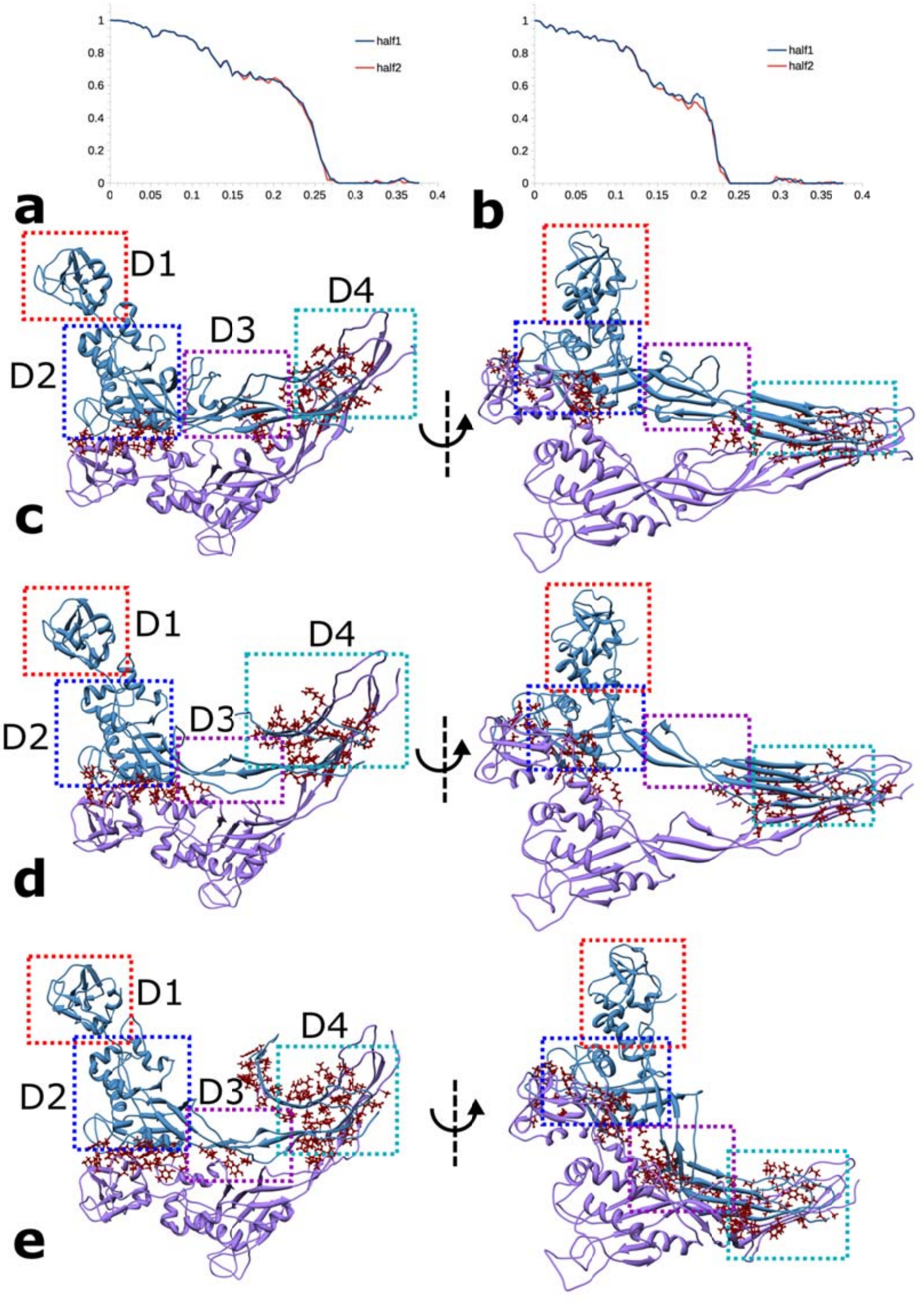

Supplementary Figure 6: Model quality assessment and inter-subunit contacts
Model quality assessment and inter-subunit contacts. **a**, FSC curves of the prepore model versus the Y221G mutant training half map (blue) and test half map (red). **b**, FSC curves of the post-prepore and quasipore models versus the K246C/E258C mutant training half map (blue) and test half map (red). **c**, Structure of two consecutive subunits of the prepore coloured in blue and purple, respectively. Residues calculated as inter-subunit contacts are shown in red as ball and stick representation. Dashed rectangles show the position of individual domains and are colour coded as in Fig. 1. **d**, Structure of two consecutive subunits of the post-prepore

as in **c**. Of note, the contact site between the two domains 4 is enlarged whilst the prestem loop gets unstructured. **e**, Structure of two consecutive subunits of the quasipore as in **a** highlighting the extensive contacts formed between subunits. In particular the β-barrel elongation provides additional contacts whilst the collapse of the structure generates new contacts between domain 2 of the purple subunit and domain 3 of the following subunit (blue).

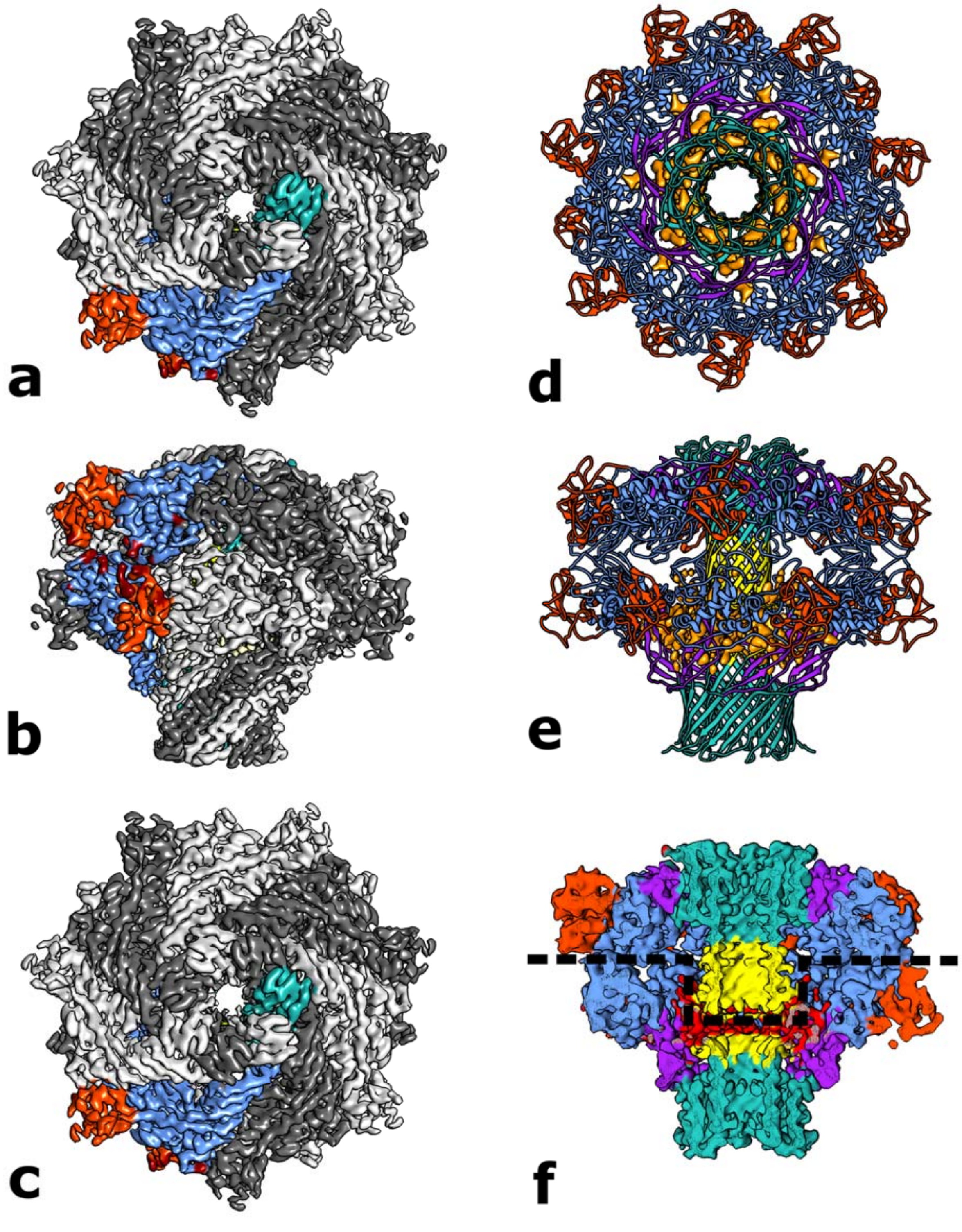

Supplementary Figure 7: K246C/E258C mutant cryo-EM map and highlights

K246C/E258C mutant cryo-EM map and highlights. **a**, Cryo-EM map top view (quasipore side) colour-coded as in Figure 1. **b**, Side view of the same map. **c**, Bottom view of the same map (post-prepore side). **d**, Top view of the fitted atomic model of the two aerolysin heptamers showing in addition in gold a low-resolution density that provides an approximate location for the flexible prestem loop. **e**, side view of the same atomic model. **f**, cut-through the cryo-EM map colour coded by domains as in 1a. Additionally low-resolution density of the prestem loop is shown in red. Dashed line separates the quasipore and post-prepore densities.

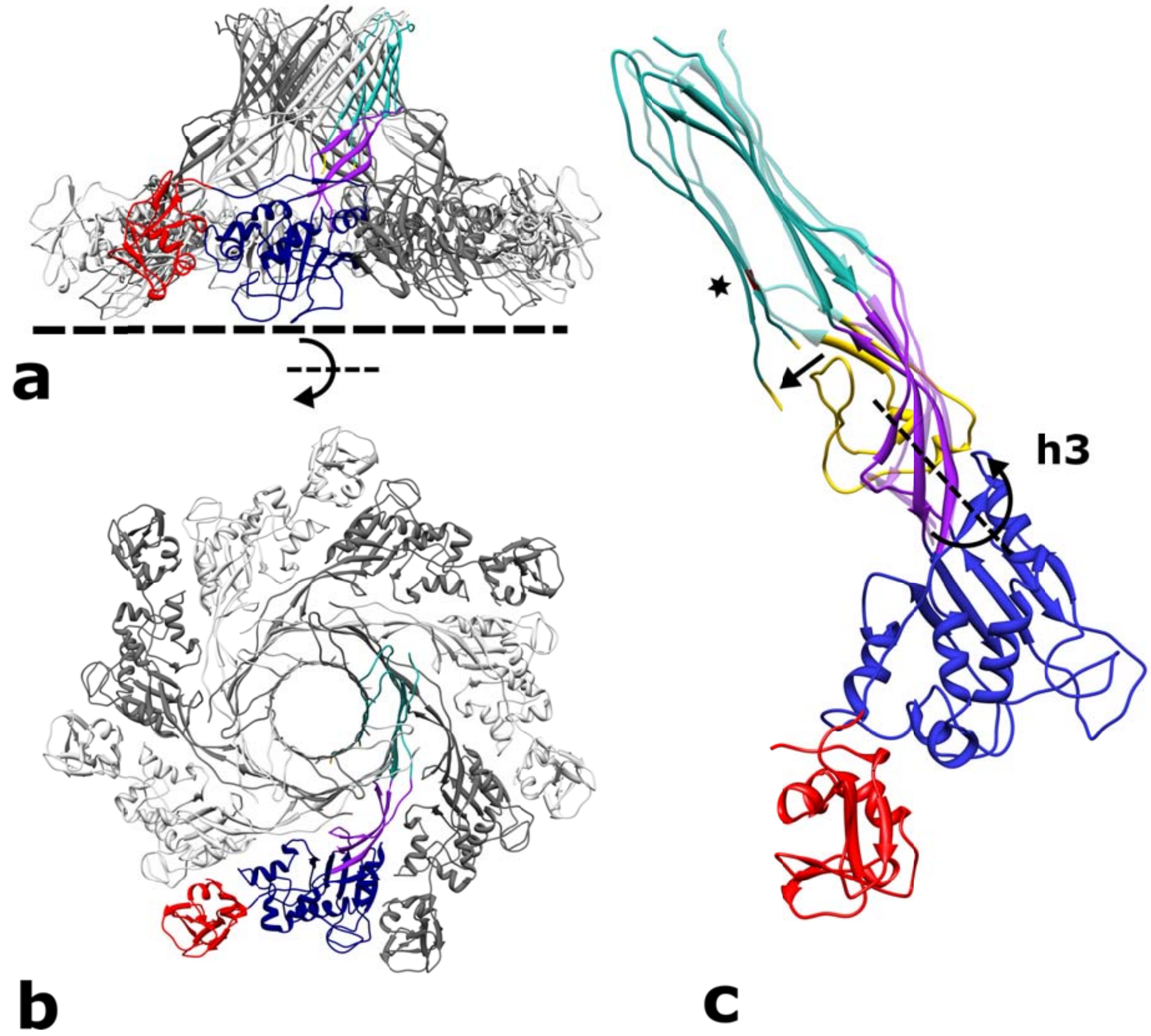

Supplementary Figure 8: Post-prepore architecture

Post-prepore architecture. **a**, Post-prepore heptamer structure side view coloured as Figure 1. Dashed line shows the position of the membrane. **b**, Post-prepore heptamer top view. **c**, Prepore to post-prepore transition. The prepore and post-prepore structures were aligned on domain 2. For clarity the prepore structure is shown semi-transparent whilst post-prepore is colour coded as in Figure 1. Domains 1 and 2 are shown for post-prepore only. The position of the Y221(G) residue is marked by a star whilst the main movement axis is shown (h3 – hinge 3). The movement of the prestem loop is shown by an arrow.

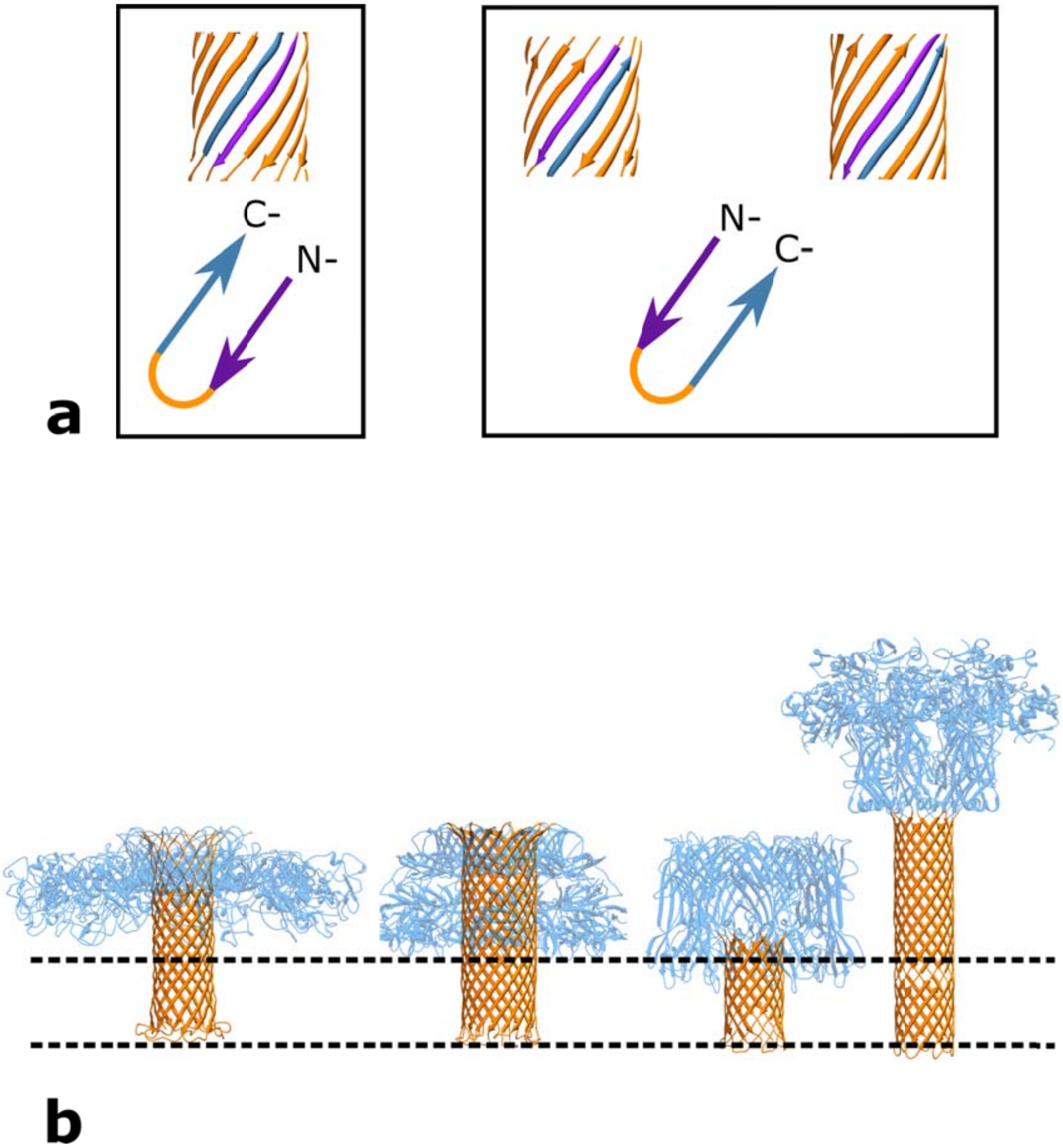

Supplementary Figure 9: PFT comparisons and strand inversion

PFT comparisons and strand inversion. **a**, Pore β-barrels seen from the outside, with the extracellular side up. Strand inversion in the aerolysin β-barrel (left box) shows an atypical conformation in which the first strand to cross the membrane to the intracellular side is positioned on the right of the β-hairpin. In comparison (right box), strand disposition for anthrax protective antigen (left, PDB 3J9C) and α-hemolysin (right, PDB 7AHL) β-barrels shows the typical left to right pattern. **b**, Comparison of the putative aerolysin fully deployed pore with other known PFT structures. From left to right, aerolysin (far left), lysenin (PDB 5GAQ), anthrax protective antigen (PDB 3J9C) and α-hemolysin (PDB 7AHL - far right). The cap domain is shown in light blue with 50% transparency for clarity. The dashed lines delineate the membrane hydrophobic core. The length of aerolysin inner β-barrel matches the length of anthrax protective antigen β-barrel and that of lysenin β-barrel.

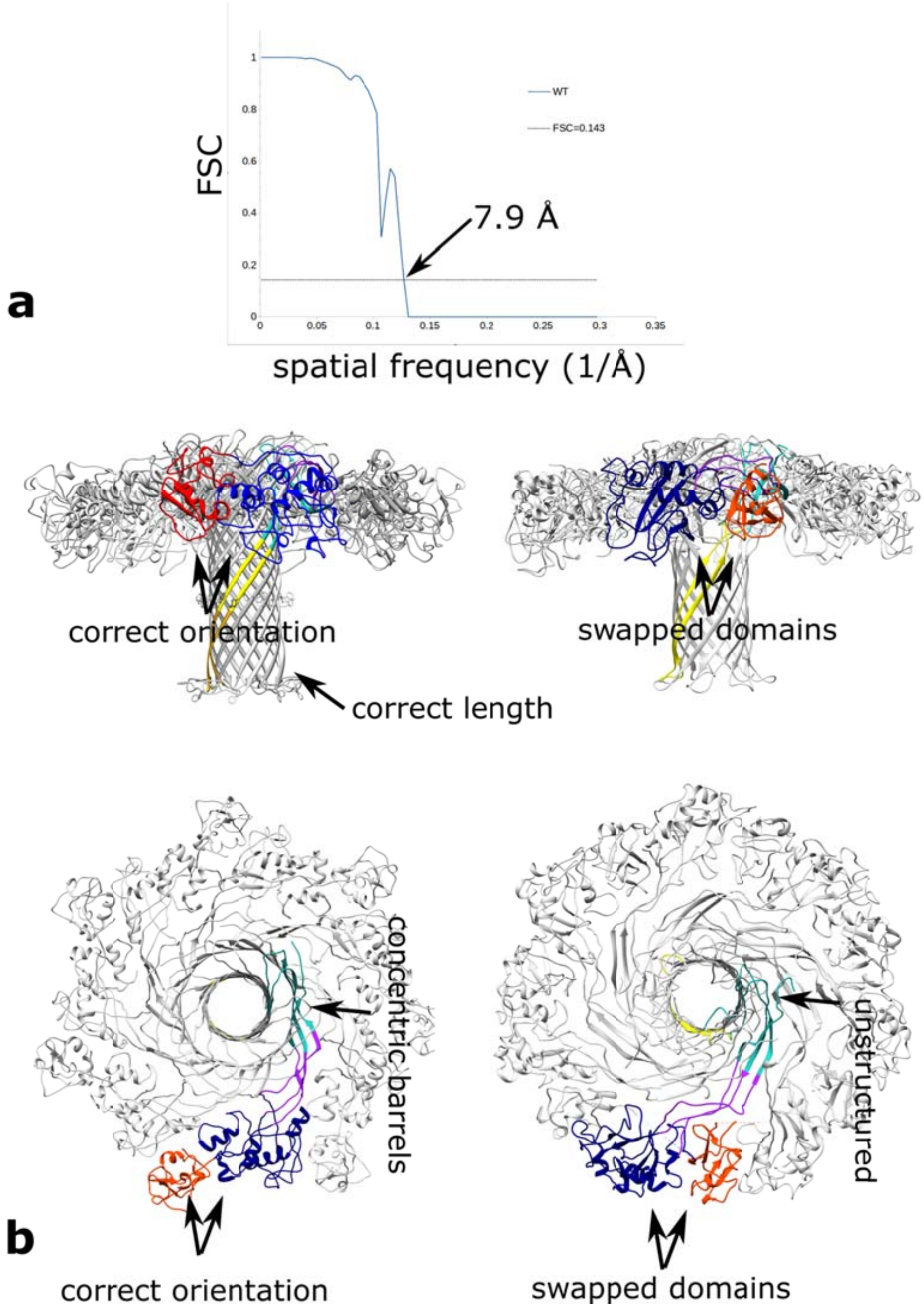

Supplementary Figure 10: FSC of the WT map and comparison of the atomic resolution structure of aerolysin heptamers and the previously published aerolysin model

FSC of the WT map and comparison between the atomic resolution structure of the aerolysin heptamers with the previously published aerolysin model[3]. **a**, gold standard FSC curve of the WT maps. The horizontal dashed line indicates the 0.143 cutoff used to estimate the global resolution of the map at 7.9 Å resolution.

b, Whilst the previous model predicted the large collapse involved in pore formation it was unable to solve key features shown in the current structure. In particular, the domains 1 and 2 of the protein were misplaced in the cap (arrows) whilst the length of the transmembrane β-barrel was underestimated. The concentric β-barrel fold (arrow – concentric barrels) was also missing from the previous model based on low resolution EM maps (arrow - unstructured).

Supplementary Note 1: comparison with previous atomic models

We previously reported atomic models for the prepore, post-prepore and quasipore conformation, which were based on low-resolution (~18 Å) cryo-EM maps coupled to dynamic integrative modelling [3]. Although these initial models correctly highlighted the large collapse taking place between the post-prepore and the quasipore conformation, and revealed that it was due to profound rearrangements in domain 3 and 4, they missed to capture some important features disclosed by our new high-resolution structures (Sup. Fig. 10). First, it missed the existence of a double concentric β-barrel fold at the outside-facing mouth of the pore, of which the inner β-barrel develops to an 87-Å long β-barrel spanning the whole length of the pore. Second, domains 1 and 2 were swapped in the old models. Third, a β-barrel strands inversion at the inner β-barrel, which is the consequence of vast reorganization needed to form the double β-barrel fold, was not predicted.

Supplementary References

1 Szczesny, P. *et al.* Extending the aerolysin family: from bacteria to vertebrates. *PLoS One* **6**, e20349 (2011).

2 Lesieur, C. *et al.* Increased stability upon heptamerization of the pore-forming toxin aerolysin. *J Biol Chem* **274**, 36722-36728 (1999).

3 Degiacomi, M. T. *et al.* Molecular assembly of the aerolysin pore reveals a swirling membrane-insertion mechanism. *Nat Chem Biol* **9**, 623-629 (2013).
